# Complete chloroplast genome sequences of Wonwhang (*Pyrus pyrifolia*) and its phylogenetic analysis

**DOI:** 10.1080/23802359.2017.1331328

**Published:** 2017-05-30

**Authors:** Ho Yong Chung, Tae-Ho Lee, Yoon-Kyeong Kim, Jung Sun Kim

**Affiliations:** a Department of Agricultural Biotechnology, National Institute of Agricultural Sciences, RDA, Jeonju, South Korea;; b Pear Research Institute, National Institute of Horticultural & Herbal Science, RDA, Naju, South Korea

**Keywords:** *Pyrus pyrifolia*, Wonwhang, chloroplast genome, phylogenetic analysis, whole-genome sequencing

## Abstract

The complete chloroplast genome of Wonwhang (BioSample SAMN05196235), *Pyrus pyrifolia*, was assembled and analyzed by *de novo* assembly using whole-genome sequencing data. The accession NC_015996 was used as a reference sequence in this study. The total chloroplast genome size of the Wonwhang was 159,922 bp in length, including a pair of inverted repeat regions (IRs) of 26,392 bp that are separated by a large single-copy region of 72,023 bp and a small single-copy region of 19,235 bp. A total of 132 genes, including 93 protein-coding genes, 31 tRNA genes and eight rRNA genes, were predicted from the chloroplast genomes. Among them, 18 genes occur in IRs, containing nine protein-coding genes, five tRNA genes and four rRNA genes. The GC content of Wonwhang chloroplast genome is 36.6%. The phylogenetic analysis with nine Rosids species and three other species revealed that Wonwhang was clustered with *Malus* genus.

Pears belong to the family of Rosaceae and have been cultivated in East Asia, Europe and North America for more than 3000 years, and are among the most important fruit crops in temperate regions (Bell 1990). Asian pears have a sweet flavour, rich juice and crisp flesh, and more excellent quality and storage-friendly than Occidental pears (Kim 2016). In South Korea, pear breeding began in the late 1920s by National Institute of Horticultural and Herbal Science (NIHHS) of Rural Development Administration (RDA). To improve the fruit quality in terms of fruit size, sugar content, flesh firmness and storage quality, many breeders frequently used interspecific hybridization (Kim 2016). Consequently, the classification of pears is very difficult because of the occurrence of both natural and artificial interspecific hybrids. Therefore, the genetic relationships and evolutionary history of Asian pears are unclear (Jiang et al. 2016). The chloroplast genomes that have a small size, conserved gene content and arrangement, and maternally inheritance are useful resources for evolutionary and phylogenetic studies (Zhang & Sodmergen 2010). In this study, we sequenced the complete chloroplast genomes of Wonwhang based on an Illumina platform.

The plant sample of Wonwhang was collected from Naju (latitude: 35° 01′25.6′′N, longitude: 126° 44′38.4′′E), Korea, and representatively identified by Dr. YK Kim of the Pear Research Station, NIHHS, RDA. Total genomic DNA was extracted from young leaves using a DNeasy Plant Mini kit (Qiagen, CA) according to the manufacturer’s instructions. Whole-genome sequencing was performed using an Illumina genome analyzer (NextSeq, Illumina, San Diego, CA) platform at LabGenomics (http://www.labgenomics.co.kr/) in Seongnam-si, Republic of Korea. Genomic libraries with 350-bp insert size were prepared by following the paired-end standard protocol recommended by the manufacturer, and each sample was tagged separately with a different index. *De novo* assembly was performed using a CLC genome assembler (4.06 beta, CLC Inc., Aarhus, Denmark) with parameters of a minimum 200–600 bp autonomously controlled overlap size, as described by Kim et al. (2015). Low-quality reads were filtered out and the remaining high-quality reads were mapped to the reference chloroplast genome of Housi (Terakami et al. 2012) with an average coverage of 80 × on the chloroplast genome. Contig alignment and scaffolding based on paired-end data resulted in a complete circular Wonwhang chloroplast genome sequence. Gene annotation was conducted using CpGAVAS (http://www.herbalgenomics.org/0506/cpgavas/analyzer/annotate). The complete chloroplast genome of Wonwhang was submitted to GenBank under the accession numbers of KX450876.

The complete chloroplast genome of Wonwhang is 159,922 bp in length, with a pair of IR regions of 26,392 bp that separate an LSC region of 72,023 bp and an SSC region of 19,235 bp. It has a GC content of 36.6% and contains 93 protein-coding genes, 31 tRNA genes and 8 rRNA genes. Among them, 18 genes occur in IRs, containing nine protein-coding genes (*ndhB*, *rpl2*, *rpl23*, *rps7*, *rps12*, *rps19*, *ycf2* and *ycf15*(x2)), five tRNA genes (*trnI-CAT*, *trnL-CAA*, *trnN-GTT*, *trnR-ACG* and *trnV-GAC*) and four rRNA genes (*rrn4.5S*, *rrn5S*, *rrn16S* and *rrn23S*). Interestingly, the *rps12* gene is triple and the *ycf15* gene is quadruple copies in chloroplast genome. Fourteen genes contain one intron and two genes (*ycf3* and *clpP*) contain two introns.

To study its phylogenetic relationships with previously reported complete chloroplast genomes, nine complete chloroplast genome sequences of Rosids and three species from other family were downloaded for analysis. Maximum-likelihood (ML) phylogenetic tree for 13 species with *Oryza sativa* as an outgroup (Figure 1). The complete chloroplast genome sequence was aligned using MAFFT v7.222 (Katoh & Standley 2013). ML analysis was performed in PHYLIP v3.695 (Felsenstein 2013) with support for branches evaluated by 1000 bootstrap replications. The phylogenetic tree showed that Wonwhang chloroplast was clustered with *Malus* genus and belonged to Rosids clade. The complete chloroplast genome of Wonwhang provides essential and important DNA molecular data for phylogenetic and evolutionary analysis for Asian pears.

**Figure 1.**
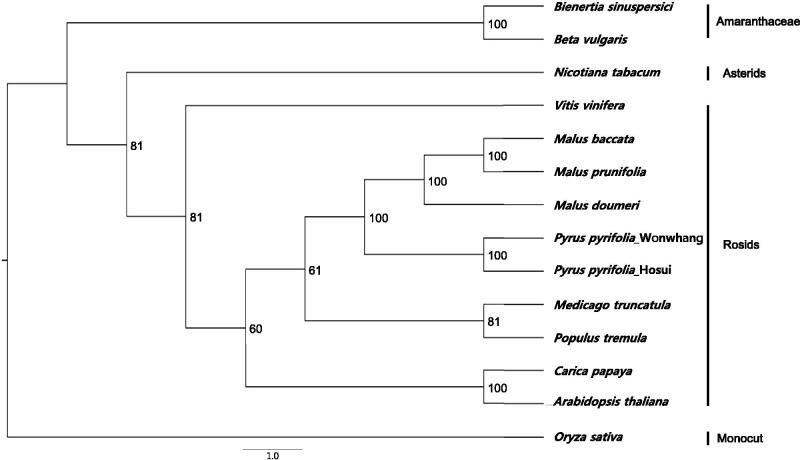
Phylogenetic relationship of the Wonwhang chloroplast genome with previously reported complete chloroplast genomes. Numbers in the nodes are the bootstrap values from 1000 replicates.
